# Bystander or active agent? Narrative analysis of Finnish chief physician career paths

**DOI:** 10.1108/LHS-01-2026-0002

**Published:** 2026-03-10

**Authors:** Sari Huikko-Tarvainen, Pasi Sajasalo, Tommi Auvinen

**Affiliations:** Faculty of Medicine, Oulu University, Oulu, Finland; School of Business and Economics, University of Jyväskylä, Jyväskylä, Finland

**Keywords:** Leadership, Chief physician, Career paths, Career capital, Narrative analysis, Healthcare

## Abstract

**Purpose:**

This study aims to examine how physicians construct their career paths into leadership and how institutional, professional and individual influences shape these trajectories. This study focuses on chief physicians to gain a deeper understanding of their lived experiences.

**Design/methodology/approach:**

Empirical data contains semistructured interviews with 15 Finnish chief physicians. Using Polkinghorne’s narrative analysis, the authors constructed two composite career narratives and examined how career capital (knowing-how, knowing-whom and knowing-why) and contextual influences shape physician leaders’ careers.

**Findings:**

Two ideal-typical narratives emerged. In the drifting narrative, physicians’ leadership emerges and develops reactively through coincidences, informal expectations or organizational necessity, with limited leadership-related knowing-why and unevenly developed knowing-how. In the bestirred agent narrative, negative leadership experiences, ethical concerns and a desire to repair dysfunctional environments motivate physicians to pursue leadership proactively, supported by deliberate development of leadership skills and networks.

**Originality/value:**

This study offers a narrative explanation of how agency and context interact in physician leadership careers and demonstrates how different configurations of career capital can lead to various pathways into leadership. This study also makes a methodological contribution: using a narrative approach to explore the lived experiences of physician leaders’ careers is a rare method in the field. In addition, the findings provide practical insights for designing leadership development and support structures that accommodate the diverse ways physicians come to lead.

## Introduction

Leadership in healthcare is essential for effective service delivery, high standards of patient care and organizational innovation (Kirkpatrick *et al.*, 2024). Physicians are often seen as well-suited for leadership, as their training emphasizes autonomous decision-making and a strong sense of professional responsibility. Hospitals led by clinically qualified physicians have been associated with improved clinical outcomes, patient satisfaction, financial performance and reduced mortality, reflecting the importance of hospital-specific management practices (Dorgan *et al.*, 2010).

Among healthcare leadership roles, the position of chief physician is particularly significant, as it combines clinical expertise with everyday administrative and strategic responsibilities (van de Riet *et al.*, 2019). Formal leaders are central to fostering employee engagement through diverse leadership functions (Günzel-Jensen *et al.*, 2018). There is also a recognized need for physician leaders, as physicians tend to prefer leadership by fellow physicians over other organizational actors (Huikko-Tarvainen, 2022; Huikko-Tarvainen *et al.*, 2021). At the same time, physicians may view nonphysician leaders (often labeled administrators) with skepticism, suspecting that their priorities may lie more in cost control or profit maximization than in patient care (Mullangi *et al.*, 2020).

Despite the recognized importance of leadership in healthcare, pathways to becoming a physician leader remain insufficiently explored, especially in terms of how institutional, professional and individual influences shape career progression. By examining the lived experiences of Finnish chief physicians and how they construct their career paths into leadership through narrative analysis, we address this gap by answering the research question:

*
RQ *. What can be learned about the mix of institutional, professional and individual influences affecting career paths of physicians progressing to leadership roles in healthcare?

In the following, we present our theoretical framework, methods, results and discussion.

## Theoretical framework

We build our theoretical framework on career capital theory and leadership development. Career capital theory, originating from work on boundaryless careers, posits that individuals accumulate different types of capital that shape their career progression and opportunities (Defillippi and Arthur, 1994). This framework distinguishes three interrelated forms of “knowing”: knowing-how (skills and expertise), knowing-whom (relationships and networks) and knowing-why (identity, values and motivation) (Inkson and Arthur, 2001; Inkson and King, 2011; Sutherland *et al.*, 2015). In physician careers, clinical expertise reflects knowing-how, professional networks and mentors reflect knowing-whom and leadership education and values underpin knowing-why. Together, these elements highlight how physician leaders draw on skills, relationships and identity to construct their career paths and how gaps in any of these may constrain movement into leadership roles (Defillippi and Arthur, 1994; Inkson and Arthur, 2001; Inkson and King, 2011; Sutherland *et al.*, 2015) The role of physician leader typically involves a hybrid task setting that combines leadership and management responsibilities with clinical work and patient care (Berghout *et al.*, 2017; Huikko-Tarvainen, 2022).

Prior clinical practice and specialist qualifications are often prerequisites for formal leadership positions, as clinical credibility is central to professional legitimacy (Huikko-Tarvainen, 2022; Spehar *et al.*, 2015). Most physicians encounter leadership responsibilities at some stage of their careers after reaching a certain hierarchical level. Even without formal titles, they often assume informal leadership roles in their practice (Huikko-Tarvainen, 2022; Maddalena, 2016). At the same time, barriers such as work–life balance challenges, strong clinical identity and systemic constraints limit the diversity and accessibility of leadership pathways (Huikko-Tarvainen *et al.*, 2021; Kippist and Fitzgerald, 2014; Sullivan *et al.*, 2022). Findings in the Australian context suggest that physicians may transition into leadership unwillingly, feeling pressured or strongly encouraged by peers and may experience themselves as underprepared while striving to maintain collegial respect through continued clinical work and balancing competing leadership and budget responsibilities (Imran *et al.*, 2021). These findings underscore how institutional arrangements, professional norms and personal circumstances may shape the lived experience of becoming a physician leader and the ways career capital is accumulated and applied.

We draw on transformational leadership theory as a complementary framework. Transformational leadership emphasizes the importance of vision, ethical orientation and interpersonal influence in mobilizing followers and fostering change (Antonakis and Day, 2018). In the physician leadership context, this perspective helps to contextualize how individual traits, values and motivations influence decisions to pursue leadership roles and how leaders seek to inspire and develop others (Lawrason *et al.*, 2023; Mason *et al.*, 2014). It also aligns with evidence that mentorship and leadership development programs may support physicians in balancing clinical and leadership responsibilities and building confidence as leaders (Lyons *et al.*, 2020). Physician leadership development programs often combine formal teaching with experiential and relational components (e.g. action learning and peer interaction) (Frich *et al.*, 2014), which can strengthen leadership knowing-how and knowing-whom, while also reinforcing leadership-oriented knowing-why.

## Data and method

We adopt a qualitative, narrative orientation to examine how the theoretical perspectives outlined above appear in our empirical data in the Finnish healthcare context. This allows us to describe and interpret emerging meanings in the lived experiences (Bryman and Bell, 2015) of our informants and to understand the institutional, professional and individual influences shaping their career paths. As our interest lies in chief physicians’ lived experiences and their meaning-making related to careers moving into leadership roles, a narrative orientation – viewing narrative as a primary form of making human experience meaningful (MacIntyre, 2007) – is well suited to our purpose.

We perceive narratives, in line with Bruner (1991) and Polkinghorne (1988), as temporally organized discursive accounts of experience that can reveal processes in the social construction of phenomena such as leadership (Boje *et al.*, 2011). Following Polkinghorne (1995), we use narrative analysis to construct composite career narratives based on the data to make lived experiences meaningful, considering the theoretical framework, and to understand how career capital accumulates and how individual and institutional influences interact in shaping leadership trajectories. In this regard, we subscribe to Sahni and Sinha’s (2016) view of narrative investigation’s aim being the interpretation of human experience.

### Data collection

The data were gathered through semistructured interviews with 50 Finnish physicians at various hierarchical levels: 14 residents/specializing physicians, 13 specialists, 8 heads of department and 15 chief physicians employed by the Central Finland Health Care District. The interview guide included themes probing physician leadership from different perspectives (career path, physician leadership features, leadership practices, support and training, work–life balance). In this article, we focus on the subset of 15 chief physicians and analyze content related to their career paths into leadership positions. We chose chief physicians because they are “in the middle of it all”: combining clinical and leadership responsibilities and positioned hierarchically between top management and subordinates, which makes this dual hybrid role a particularly interesting balancing act to study (Berghout *et al.*, 2017; Huikko-Tarvainen, 2022).

Participants were invited via an internal email to the Health Care District’s physicians. They were informed about the purpose of the study, voluntary participation and their right to withdraw or refuse the use of their data at any time. Consent for participation and research use of the data was obtained at the beginning of each interview. The interviews were conducted between April and June 2017 and between July and August 2018, primarily in the informants’ offices. All interviews were digitally recorded and transcribed *verbatim*. The resulting corpus consists of 195 A4 pages of text (Calibri, 12-point, single-spaced), totaling 72,844 words. Table 1 summarizes the interview data by informant.

**Table 1.** Description of the data

Informant	Sex	Transcript (pages)	Word count
1_N	Female	4	1,949
2_M	Male	5	2,041
4_M	Male	8	4,187
5_M	Male	12	4,661
7_N	Female	12	6,284
8_M	Male	8	2,978
L_5	Male	14	4,187
L13	Male	8	3,209
L16	Male	6	2,362
L21	Male	11	4,454
L43	Male	16	5,118
L34	Male	36	10,911
L36	Female	20	7,418
L47	Male	16	6,414
L15	Male	19	6,671
Total		195	72,844

**Source(s):** Authors’ own work

### Analysis of the data

Following Polkinghorne’s (1995) approach to narrative analysis (narrative configuration), we constructed composite narratives that capture chief physicians’ meaning-making about their career paths into physician leadership. The narratives contain the experiences of several informants but following Polkinghorne (1995) we use the first person in a composite narrative because it most authentically conveys the participants’ lived experience by presenting the synthesized storyline from within the perspective of the composite protagonist.

Our analysis proceeded in two steps. First, we repeatedly read the interview transcripts to become familiar with each account and to map key events and turning points in temporal order, with particular attention to how interviewees linked experiences to the outcome of becoming a chief physician. During this interpretive reading, a recurring contrast emerged between accounts characterized by initial unwillingness or avoidance and those marked by an active, change-motivated orientation toward leadership. Second, guided by this sensitizing contrast, we configured the most salient and recurrent events, influences and motivations into two information-rich, re-storied composite career-path narratives. These narratives synthesize multiple interviews rather than reproduce any single account, allowing us to foreground common patterns across informants while preserving complexity and variation in how lived experience – leadership trajectories – unfolded.

### Trustworthiness and ethics

To enhance trustworthiness, we relied on investigator triangulation by involving three researchers in the analysis, independently reading the transcripts, generating initial observations and then comparing and negotiating interpretations (Eriksson and Kovalainen, 2008). An audit trail of analytic decisions, narrative drafts and memos was maintained to document the development of the analysis. To support assessment of transferability, we provide a thick description of the study context and the two composite narratives, enabling readers to judge the applicability of the findings to comparable healthcare settings (Lincoln and Guba, 1985; Loh, 2013). We followed national and international ethical standards for nonmedical research involving human participants, in line with the guidelines of the Finnish National Board on Research Integrity and EU data protection regulations (Finnish National Board on Research Integrity TENK guidelines, 2019). The Central Finland Health Care District granted approval for the study according to its policies.

## Results

Next, we present two composite career path narratives, *drifting* and *bestirred agent*, as the main analytic outcome of the study, constructed from the lived experience accounts of 15 chief physicians narrated in the first person. Selected excerpts are provided as illustrative evidence, showing how the narratives are grounded in physicians’ own accounts. Each narrative illustrates how institutional, professional and individual influences shape progression into chief physician roles and how these influences accumulate as career capital: knowing-how (skills/credibility), knowing-whom (networks/mentors) and knowing-why (identity/purpose). These patterns are then summarized in Table 4.

### *Drifting narrative – unwillingly ending up in a leadership* role

This narrative combines thematic content related to arbitrariness and highlights how the career path to a physician leadership position is constructed on lived experiences of randomness, drifting and the influence of other actors, rather than on systematic, self-driven plans. The underlying logic is unwillingness and avoidance of leadership roles, as reflected in the lived experiences of multiple chief physicians:

I was supposed to become, first and foremost, medical practitioner, not leader. The original plan was clear: clinical work and patients, perhaps specialization in a well-defined field of medicine that would anchor my professional identity. I studied, specialized, and did a lot of clinical work, expecting a linear career of deepening expertise within my chosen specialty.

Over time, however, organizational needs began to rewrite my original plan. Vacant posts, sudden departures of colleagues, and the lack of other willing candidates created situations where someone “had to” take responsibility. I happened to be around when a quick fix was needed for the organization to function. Gradually, I drifted into positions where I was responsible for others, not because this aligned with my aspirations, but because the work just had to be done. Someone saw me as the obvious solution.

Leadership did not arise from my own career ambitions but from practical workplace demands. Clinical work was pushed aside, not by my personal choice but by organizational necessity. I found myself in meetings I had never planned to attend. I was dealing with budgets, personnel issues, and strategic decisions I had never imagined handling. I felt badly unprepared. With no formal leadership education and no one handing me even a basic textbook on leadership, I had to learn the ropes by “the hard way” in my everyday organizational life – kind of on the side. My leadership was therefore constant improvisation and patchwork: negotiating, listening, trying to take my space, and acting without a chance to rehearse or prepare for the job.

My next career moves were often greatly swayed by “others”. A supervisor’s insistence pushed me apply for a position, or strong external expectations redirected my path, even though I would have preferred remaining a clinician as I had originally intended. In retrospect, I didn’t recognize myself as the author of my career, but as a narrator weaving together coincidences, demands, and small turning points that pushed me into leadership.

As the drifting narrative shows, physician leadership thus became an unintentional role, yet it is intertwined with the lived experience as an inseparable part. Careers were marked by uncertainty, side paths and surprises that eventually shaped identities as physician leaders and left leadership feeling like an organizational necessity rather than a consciously chosen pursuit. Excerpts in Table 2 anchor the drifting narrative in institutional, professional and individual influence layers.

**Table 2.** Illustrative excerpts: drifting narrative

Drifting narrative	Illustrative excerpts
Institutional influences: vacancies, ad hoc appointments, learning by doing	I had been working just maybe a couple of years, so I happened to be the doctor in charge of a health center, so that’s just how it goes. (L43); We have not necessarily taken [leadership] courses, but learned leadership the hard way, by trial and error. (L15)
Professional influences: clinical primacy, identity pull toward patient work	I operate four days a week. Any “leadership” must fit around clinical work. (4_M)
Individual influences: low planning, avoidance, reactive agency	I try to enable people to have influence on their work… but I’m not a systematic leader. There’s room for improvement for me. (2_M)

**Source(s):** Authors’ own work

The career path into leadership can be constructed as a sequence of organizational contingencies and informal expectations rather than as a conscious choice. Physicians describe stepping into leadership because posts were vacant, someone had to take responsibility or others expected them to do so. Leadership knowing-how is largely developed through learning “the hard way,” and leadership knowing-why remains weak and conflicted. These patterns resonate with earlier studies that depict physician leadership as a by-product of clinical careers and organizational necessity rather than a planned trajectory (Imran *et al.*, 2021; Spehar *et al.*, 2017; Spurgeon *et al.*, 2015). This represents a dilemma: without conscious leadership identity building, the transition to leadership may fall short, affecting the quality of leadership and the well-being of medical leaders (Spehar *et al.*, 2017), not to mention their followers.

### Bestirred agent narrative – stepping up to end a vicious cycle

This narrative combines thematic content related to active agency and portrays the career path to physician leadership as a corrective effort: an ethically motivated choice constructed on lived experiences of bad leadership and an ensuing commitment to change working conditions for the benefit of the profession. The underlying logic is responsibility rather than ambition:

I began as an ordinary physician, not a career missile or power-seeker, but a committed professional with a desire to do my work well. My days were filled with patients, consultations and the usual rush of clinical practice. Over time, however, I developed a nagging sense that something was wrong. Not in the core of patient care, but in what surrounds it – leadership. More and more decisions were just handed down to me from above without explanation. Changes affecting my work were put into effect without discussion or the slightest consultation. Leaders started to appear distant or even hostile to me and my colleagues. Witnessing how exhausted my colleagues grew with the situation and hearing their repeated sighs of frustration, I came to see these problems as structural rather than incidental.

I had not originally planned to become a leader of my colleagues. Yet when I was confronted with the question of who would change this reality, it hit me. I felt like I just couldn’t remain a bystander any longer. Having seen employees losing trust in their leaders, I decided I didn’t want to watch passively the situation deteriorate further. Instead, I chose to take role in changing things, be an active agent. I accepted that if “someone” had to step up, it would be me. This conviction led me to seek leadership education and apply for leadership positions as a hands-on way to change the status quo that, in my view, had persisted far too long.

Assuming a leadership role, to me, was about responsibility: to myself, to my colleagues and to the profession. I had lived through the consequences of missing, misplaced and poor leadership and was determined to become the kind of leader I had rarely experienced earlier in my career. My conviction was to be approachable, fair, and unafraid of criticism. I aspired to build work environments where decision-making rested on openness, transparency, and willingness to listen, even to critical voices. I took colleagues’ critique, not as a threat to my authority, but as a sign of genuine care and commitment to shared goals. I actively created room for discussion, even those difficult conversations, to ensure that people around me felt heard and empowered to influence the conditions of their daily work.

As the bestirred agent narrative indicates, the physicians felt that they were less supervisors than mediators and enablers. Their ambition was to listen sincerely and to relate to those they led as colleagues. Drawing on their lived experience of bad leadership, they concluded that what followers needed was not increased control or monitoring, but collegial encounters, support and protection from excessive workload. They kept close to clinical realities, remaining aware of what was happening in emergency rooms, offices and coffee rooms. Being present, visible and accessible to physician colleagues was understood as an ethical act grounded in shared professional values.

Their leadership was, therefore, primarily a form of service. It was born from awakening to the pain of dysfunctional leadership, and it developed into an active effort to transform that collectively felt pain into hope. By responding to colleagues’ unspoken wishes, “if only we did not have to put up with this,” they sought to demonstrate, in practice, that physician leadership could be done differently.

Leadership thus emerges as an active pursuit to repair dysfunctional climates, where ethical purpose crystallizes into agency. These physicians seek educational programs and roles that enable transparent communication, collegial support and sustainable workloads. Their trajectories reflect a strong knowing-why that motivates deliberate development of leadership knowing-how and purposeful cultivation of knowing-whom. The following excerpts in Table 3 anchor the bestirred agent narrative in institutional, professional and individual influence layers.

**Table 3.** Illustrative excerpts: bestirred agent narrative

Bestirred agent narrative	Illustrative excerpts
Institutional influences: reform intent triggered by authoritarian or unclear leadership	There were many problems; leadership blamed staff; consultants told us we were wrong and the leader was right… I thought, without change, this won’t work. I took leadership training, and when the chief post opened – I applied. (L47) [Getting on the job], the healthcare district director just said to me, “get leading” – bad leadership if you ask me. Good leadership should be clear: a chain from top to bottom about what, how, why. (L34)
Professional influences: collegial care, transparency and legitimacy	Long careers bring continuity… I wanted to change leadership toward openness and dialogue not to lose good people. (L47)
Individual influences: ethical conviction, boundary-setting, identity work, active agency	There was too just much work… people were near burnout… I had to do something… a unit head role came up, so I took it – and liked it. (5_M)

**Source(s):** Authors’ own work

As the narrative demonstrates, experiences of poor or authoritarian leadership, heavy workloads and colleagues’ exhaustion can act as turning points that generate a sense of responsibility to lead differently. Physicians construct leadership as a way to repair dysfunctional climates and protect colleagues, which strengthens leadership knowing-why and motivates deliberate investment in knowing-how and knowing-whom. This aligns with earlier findings on negative leadership experiences as powerful “negative learning” events that spur efforts to develop better leadership practices (Spehar *et al.*, 2017) and with work emphasizing everyday presence, listening and participation in physician leadership (Kuivalainen *et al.*, 2024).

The two composite narratives above highlight how institutional, professional and individual influences shape chief physicians’ leadership careers in distinct ways. To summarize these patterns, Table 4 compares how these influence layers are configured as forms of career capital: knowing-how, knowing-whom and knowing-why in the drifting and bestirred agent paths. The table condenses what is already visible in the narratives into a comparative format.

**Table 4.** Influence layers and career capital mechanisms in the two narratives

Influence layer	Drifting narrative (how it shows)	Career capital in drifting	Bestirred agent narrative (how it shows)	Career capital in bestirred agent
Institutional	Vacancies/ad hoc appointments, learning by doing	Knowing-how: improvised; knowing-whom: via supervisor; knowing-why: thin	Dysfunctions motivate reform, program access, seeking opportunity when posts open	Knowing-why: strong; knowing-how: intentional; knowing-whom: cultivated
Professional	Clinical primacy, skepticism of “pure” administrative roles	Knowing-how: anchored in clinical; knowing-whom: limited; knowing-why: weak	Collegial care and transparency valued	Knowing-why: aligned with ethics; knowing-whom: broadened; knowing-how: extends
Individual	Avoidance: reactive agency	Knowing-why: conflicted; knowing-how: piecemeal; knowing-whom: situational	Identity shift: purposeful agency, boundary-setting	Knowing-why: robust; knowing-how: intentional; knowing-whom: actively built

**Source(s):** Authors’ own work

In the drifting narrative, institutional influences are dominated by vacancies, informal succession and *ad hoc* appointments that draw physicians into leadership without clear preparation or transparent recruitment. As summarized in Table 4, leadership knowing-how develops mainly through improvisation and on-the-job learning, knowing-whom remains relatively narrow and tied to local hierarchies and leadership knowing-why is weak and often ambivalent. Leadership is constructed as an organizational necessity, while clinical identity continues to dominate.

In the bestirred agent narrative, institutional influences work as catalysts rather than constraints. Experiences of inadequate or authoritarian leadership and unclear structures motivate physicians to seek leadership education and positions to improve conditions. Knowing-why is strong and grounded in ethical concerns for colleagues and patients, knowing-how is developed deliberately through formal and informal learning and knowing-whom extends beyond immediate clinical teams to wider organizational and interprofessional networks. Overall, Table 4 demonstrates how similar settings can produce contrasting configurations of career capital: reactive and uneven in drifting careers, intentional and ethically anchored in bestirred careers, providing a basis for the discussion that follows.

## Discussion

This study examined how chief physicians construct their career paths into leadership and what these constructions reveal about the interplay of institutional, professional and individual influences in the accumulation of leadership-related career capital. Using narrative analysis (MacIntyre, 2007; Polkinghorne, 1995) of interviews with Finnish chief physicians, we identified two ideal-typical career narratives: a drifting narrative, in which physicians find themselves in leadership through vacancies, *ad hoc* decisions and external expectations and a bestirred agent narrative, in which physicians deliberately move into leadership to address perceived problems and injustices in their work environment.

In the drifting narrative, leadership emerges as an unplanned side path to clinical work. Chief physicians construct their careers as sequences of contingencies in which they step into leadership because “someone had to,” because no one else applied, or because they were already doing many of the tasks. A similar accidental, or serendipitous, path to leadership was noted by Walsh and Johnston (2025). While their context was volunteer organizations, the path appeared similar: unplanned and accidental (Walsh and Johnston, 2025). In the physician leadership context, institutional practices, such as informal succession, temporary substitutions turning into permanent roles and limited formal recruitment procedures shape these unplanned trajectories, echoing earlier findings that physician leadership careers are often nonlinear, unclear and reactive (Imran *et al.*, 2021; Spehar *et al.*, 2017; Spurgeon *et al.*, 2015; Tuononen *et al.*, 2018). Professional norms that prioritize clinical expertise and treat leadership tasks as secondary further support a situation where leadership appears as an obligation rather than a chosen career goal (Huikko-Tarvainen, 2022; Kippist and Fitzgerald, 2014; Sullivan *et al.*, 2022). From a career capital perspective, these trajectories are characterized by strong clinical knowing-how and established clinical knowing-whom, but relatively thin leadership-related knowing-why (Defillippi and Arthur, 1994; Inkson and Arthur, 2001; Sutherland *et al.*, 2015). Leadership knowing-how is accumulated retrospectively through “learning the hard way,” informal mentoring and on-the-job trial and error rather than through systematically designed development pathways (Frich *et al.*, 2014; Lyons *et al.*, 2020).

In contrast, the bestirred agent narrative emphasizes strong personal agency and ethical motivation. Here, lived experiences of poor or authoritarian leadership, dysfunctional work environments and perceived threats to patient care or professional values act as turning points that “stir” physicians to step forward. Leadership becomes a means to repair and improve the system. These physicians describe actively seeking leadership training, feedback and broader networks to gain the competence and legitimacy needed to implement change, in line with findings on negative leadership experiences as catalysts for taking responsibility to develop better practices (Kuivalainen *et al.*, 2024; Spehar *et al.*, 2017). In career capital terms, bestirred agents are marked by a robust leadership-oriented knowing-why that is closely tied to professional ethics and care for colleagues (MacIntyre, 2007; Schein, 1978). This strong sense of purpose drives deliberate investment in leadership knowing-how and systematic expansion of knowing-whom beyond immediate clinical colleagues to include organizational and interprofessional networks (Frich *et al.*, 2014; Lyons *et al.*, 2020).

Taken together, the two narratives extend research on physician leadership and hybrid physician–manager roles in three ways. First, they make explicit the more passive and more active forms of agency in physician leadership careers, complementing work on hybrid managers and identity work in healthcare (Berghout *et al.*, 2017; Bresnen *et al.*, 2019; Spehar *et al.*, 2015). Second, they show how career capital accumulates differently across these paths: in drifting trajectories, leadership capital is built reactively and unevenly, whereas in bestirred trajectories it is built more intentionally and anchored in a strong sense of purpose (Defillippi and Arthur, 1994; Inkson and King, 2011). Third, our findings underline the role of negative leadership experiences and workplace problems in motivating some physicians to take up leadership roles, adding nuance to earlier emphases on positive role models and formal opportunities (Frich *et al.*, 2014; Kuivalainen *et al.*, 2024; Lyons *et al.*, 2020; Spehar *et al.*, 2017).

From a practical viewpoint, the distinction between drifting and bestirred career paths suggests that organizations should not treat physician leaders as a homogeneous group. For physicians likely to drift into leadership, organizations could make leadership a more visible and legitimate career option earlier in professional life, for example, through career discussions, shadowing and introductory leadership programs targeted at senior clinicians (Fairchild *et al.*, 2004; Lyons *et al.*, 2020). Clarifying expectations for the chief physician role and offering structured onboarding, mentoring and peer support can help transform reactive leadership into more purposeful leadership and strengthen leadership-related career capital (Lyons *et al.*, 2020; Sullivan *et al.*, 2022). For bestirred agents, organizations should recognize both their potential as change agents and the emotional demands they face. Providing formal authority commensurate with responsibilities, ensuring access to supportive leadership networks and legitimizing efforts to challenge existing practices are crucial to sustaining their engagement and well-being (Huikko-Tarvainen, 2022; Spurgeon *et al.*, 2015; Sullivan *et al.*, 2022).

This study is based on narrative interviews with 15 chief physicians in a single national context and the findings should be interpreted with this scope in mind. The sample is skewed toward male physicians, limiting analysis of gendered aspects of leadership careers. The data were collected in 2017–2018, before some recent healthcare reforms and the COVID-19 pandemic; subsequent system changes may have created new pressures and opportunities for physician leadership (Kirkpatrick *et al.*, 2024). Narrative approach, focusing on researcher-constructed composite narratives, allowed us to foreground meaning-making and agency in physician leadership careers (Bruner, 1991; Czarniawska, 1998; Vaara *et al.*, 2016), but it abstracts from individual life histories and cannot capture all possible trajectories. Future research could examine drifting and bestirred narratives in other specialties and countries, follow career constructions longitudinally, or combine narrative analysis with quantitative data on career outcomes.

## Conclusion

This study shows how chief physicians construct their paths into leadership between drifting and bestirred agency. In drifting trajectories, physicians enter leadership in response to organizational needs and external expectations, with limited prior intention or preparation. In bestirred agent trajectories, they deliberately pursue leadership roles to address perceived injustices, dysfunctional practices or threats to professional values. By linking these trajectories to knowing-how, knowing-whom and knowing-why, we show that physician leadership careers are not only about acquiring leadership skills or positions but also about developing a meaningful sense of purpose and relational embeddedness as leaders (Defillippi and Arthur, 1994; Inkson and Arthur, 2001; Inkson and King, 2011; Sutherland *et al.*, 2015). Taken together, our findings suggest that healthcare organizations should not assume that physicians naturally grow into effective leaders through clinical seniority alone, but should design development structures that support both those who drift and those who are bestirred to step up into leadership, to enhance the quality and sustainability of physician leadership for the benefit of medical staff and ultimately, the patients and healthcare systems (Huikko-Tarvainen, 2022; Kirkpatrick *et al.*, 2024).

## Data Availability

The data that support the findings of this study are available from the corresponding author upon reasonable request.
